# Unified theory of the anomalous and topological Hall effects with phase-space Berry curvatures

**DOI:** 10.1126/sciadv.abq2765

**Published:** 2022-11-09

**Authors:** Nishchhal Verma, Zachariah Addison, Mohit Randeria

**Affiliations:** Department of Physics, The Ohio State University, 191 W Woodruff Ave., Columbus, OH 43210, USA.

## Abstract

Spontaneously broken time-reversal symmetry in magnetic materials leads to a Hall response, with a nonzero voltage transverse to an applied current, even in the absence of external magnetic fields. It is common to analyze the Hall resistivity of chiral magnets as the sum of two terms: an anomalous Hall effect arising from spin-orbit coupling and a topological Hall signal coming from skyrmions, which are topologically nontrivial spin textures. The theoretical justification for such a decomposition has long remained an open problem. Using a controlled semiclassical approach that includes all phase-space Berry curvatures, we show that the solution of the Boltzmann equation leads to a Hall resistivity that is just the sum of an anomalous term arising from momentum-space curvature and a topological term related to the real-space curvature. We also present numerically exact results from a Kubo formalism that complement the semiclassical approach.

## Teaser

Hall effects in chiral magnets are described in terms of momentum-space and real-space Berry curvatures.

## INTRODUCTION

Skyrmions in chiral magnets are topological spin textures (*1*) that are of great interest both for their fundamental properties and their technological promise as new platforms for information storage and computation. These textures can be directly imaged using a variety of techniques, but their simplest experimental signature is in electrical transport. The “topological charge” density of skyrmions affects the flow of electrons via a real-space Berry curvature and leads to the topological Hall effect (THE). This, however, is only part of the measured Hall resistivity data in chiral magnets.

Hall data in these systems are routinely analyzed as a sum of two nontrivial contributions, an anomalous Hall effect (AHE) that exists in the presence of a net magnetization and the THE described above, in addition to the ordinary Hall response proportional to the external field. This has become the standard way of interpreting Hall data in skyrmion materials ranging from conducting B20 crystals (*2*–*4*) and thin films (*5*–*7*) to heavy metal/magnetic insulator bilayers (*8*, *9*).

Despite much effort, however, a rigorous theoretical justification for expressing the total Hall resistivity as the sum of these contributions has been lacking thus far. Here, we demonstrate that the Hall resistivity can be written as the sum of the AHE and THE within a controlled calculation. While our final result is simple, its derivation involves a complex route: developing a semiclassical formalism that takes into account all phase-space Berry curvature effects on an equal footing, including **r-**space, **k-**space, and mixed curvatures, and solving the Boltzmann transport equation in a controlled fashion.

To put our work in perspective, we note that existing theories of the AHE and THE are distinct, and efforts to combine them have not led to simple predictions in the past. Karplus and Luttinger (*10*) identified the importance of spin-orbit coupling (SOC) in the AHE. Their analysis is now best understood in terms of an anomalous velocity of conduction electrons arising from the **k**-space Berry curvature of the band structure (*11*–*13*) in a system with a net magnetization. Additional extrinsic contributions to the AHE arise from scattering processes (*14*, *15*) in the presence of SOC. However, it is well established that the intrinsic **k**-space Berry curvature effect dominates over the extrinsic scattering contribution in many AHE experiments (*12*, *16*). We thus focus below only on the intrinsic part of the AHE.

Theories of THE, on the other hand, are based on a real-space Berry curvature effect that arises from the coupling of the conduction electrons to emergent electromagnetic field (*1*, *17*, *18*) of topological spin textures. There has been much work on refining these theories (*19*–*23*) and on analyzing the effects of SOC (*24*–*32*) on the conduction electrons interacting with skyrmions. In particular, Bouaziz *et al.* (*32*) have analyzed the scattering of electrons from a single skyrmion, without including the anomalous velocity arising from **k**-space Berry curvature, and have found a noncollinear Hall contribution in addition to THE and the extrinsic AHE.

We emphasize, however, that a single theory that incorporates both real- and momentum-space Berry curvature effects to calculate transport has remained elusive. We present here a unified theory of THE and the intrinsic AHE in chiral magnets that accomplishes this goal.

## RESULTS

The semiclassical approach has been tremendously successful in understanding electronic transport in metals (*33*). It is the natural avenue to study the effects of both **r**-space and **k**-space Berry curvatures on an equal footing, which also requires the inclusion of mixed phase-space curvatures in our analysis (*13*, *34*). The derivation of the semiclassical equations of motion (*13*) of suitably defined wave packets in phase space is rigorously justified when there is a separation of length scales: Both the scale *L*_s_ on which the spin texture varies and the mean-free path 𝓁 from impurity scattering must be much larger than the microscopic-scale $k_{F}^{-1}$ of order the lattice spacing *a*.

To determine the Hall resistivity, we solve the Boltzmann equation to linear order in the electric field in the presence of all phase-space curvatures and real- and momentum-space derivatives of the semiclassical energy eigenvalues. This solution is simplest in the regime $L_{s}\gg \ell \gg k_{F}^{-1}≃a$. In addition, we exploit the fact that in the materials of interest, the SOC is weak with λ ≪ *E*_F_, the Fermi energy. Systematically classifying the resulting array of terms in the solution of the Boltzmann equation in powers of the small parameters λ/*E*_F_ and 𝓁/*L*_s_ and extracting the leading contributions, we find that

$$\rho_{\mathrm{xy}}=\rho_{\mathrm{xy}}^{\text{AHE}}+\rho_{\mathrm{xy}}^{\text{THE}}+{\delta \rho }_{\mathrm{xy}}$$ (1)

**Fig. 1.**
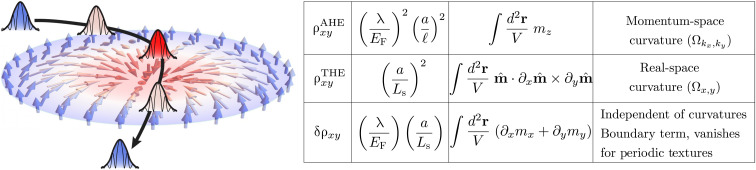
Summary of results. The semiclassical wave packet follows the texture and is influenced by real-space Berry curvature arising from the presence of skyrmions, in addition to the anomalous velocity that it acquires from an external electric field and momentum-space Berry curvature. Our results are obtained in the regime where spin texture length scale *L*_s_ ≫ mean-free path *l* ≫ *a*, the lattice spacing, and weak SOC λ ≪ *E*_F_, the Fermi energy. The table summarizes the three contributions to ρ*_xy_*, their scaling with these parameters, their dependence on the magnetic texture $\hat{m}(r)$, and their relation to Berry curvatures. Mixed momentum- and real-space curvatures contribute to the Hall resistivity at higher order in (λ/*E*_F_) and (*a*/*L*_s_).

Our semiclassical results are summarized in the table in Fig. 1, where we show how each term depends (i) on the small parameters that control our calculation, (ii) on the spatially varying magnetization $M=M_{s}\;\hat{m}(r)$, and (iii) on the Berry curvatures. While the first two terms represent the AHE and THE, respectively, the correction term δρ*_xy_* is a curvature-independent boundary contribution proportional to the vorticity of the local electronic velocity field. It vanishes when the spin texture is periodic, e.g., a skyrmion crystal, and is negligible for a disordered skyrmion array in the thermodynamic limit. We show that the mixed curvatures contribute to the Hall resistivity at higher order in the small parameters (λ/*E*_F_) and (*a*/*L*_s_) than the terms shown in Fig. 1.

Last, we also present results using the Kubo formula in the opposite regime, where $\ell \gg L_{s}≳k_{F}^{-1}≃a$. We focus on a disorder-free system with 𝓁 = ∞, use exact diagonalization in the magnetic unit cell of a skyrmion crystal, and compute the total Hall conductivity using the TKNN (Thouless, Kohomoto, Nightingale and den Nijs) formula (*35*) in the magnetic Brillouin zone, which includes the effects of both the anomalous velocity and of the skyrmion topological charge density.

These numerically exact results do not allow a simple partitioning of the Hall effect into well-defined separate contributions; nevertheless, we can get qualitative insights into aspects of these results by comparing them with the analytical results in the semiclassical regime.

Chiral magnets host skyrmion textures (*36*) with length scales 10 ≲ *L*_s_ ≲ 500 nm, while 10 ≲ *k_F_*𝓁 ≲ 10^3^, so that the mean-free path is in the range of 1 ≲ 𝓁 ≲ 100 nm. Thus, all regimes of 𝓁/*L*_s_ can be relevant depending on the material. We focus here on the two limits *a* ≪ 𝓁 ≪ *L*_s_ and *a* ≲ *L*_s_ ≪ 𝓁 where we have control on the analysis, semiclassics in the former and exact diagonalization in the latter.

### Model

We analyze a minimal Hamiltonian for studying the confluence of AHEs and THEs. It can either arise from an “s-d model” of itinerant electrons interacting with local moments in a metallic magnet with SOC or serve as a phenomenological model for conduction electrons in a metal proximate to a magnetic insulator where broken inversion symmetry induces interfacial SOC. Our main conclusions are independent of the form of the electronic dispersion or of the Rashba SOC (see Supplementary Text for more detail). For concreteness and simplicity, we consider the simple two-dimensional (2D) Hamiltonian

$$\hat{H}=\frac{{\hat{p}}^{2}}{2m}+\frac{a\lambda }{\hbar }(\hat{p}\;\times \;\hat{z})\cdot \sigma -J\;\hat{m}(\hat{r})\cdot \sigma +{\hat{H}}_{\text{imp}}$$ (2)

which describes itinerant electrons of mass *m* and Rashba SOC λ whose spin **σ** is coupled to a given magnetic texture $M=M_{s}\;\hat{m}(r)$ via an exchange interaction *J*. The elastic scattering of electrons off a disorder potential is described by ${\hat{H}}_{\text{imp}}$ and leads to a mean-free path $\ell \gg k_{F}^{-1}$. The small hats denote unit vectors, and the wide hats denote quantum mechanical operators. On the basis of the separation of time scales associated with the itinerant electrons and the dynamics of spins in the texture, we assume that the texture is static. The model has three energy scales (the Fermi energy *E*_F_, SOC λ, and exchange coupling *J*) and three length scales [the interparticle spacing $k_{F}^{-1}$ (≈*a*, the lattice spacing), the mean-free path 𝓁, and the length scale *L*_s_ associated to the spatial variations of the magnetic texture]. We will focus on the weak SOC regime λ ≪ *J*, *E*_F_, relevant for experiments.

### Semiclassical equations of motion

Let us first focus on the semiclassical regime $L_{s}\gg k_{F}^{-1}$. To analyze the dynamics of electron wave packets in phase space **ξ** = (*x*, *y*, *k_x_*, *k_y_*), we follow the standard prescription (*13*) to construct the semiclassical Hamiltonian

$$H(\xi )=\frac{\hbar^{2}k^{2}}{2m}+d(\xi )\cdot \sigma$$ (3)

where $d(\xi )=a\lambda (k\;\times \;\hat{z})-J\hat{m}(r)$ captures the quantum mechanical nature of the spin. The semiclassical eigenenergies are ℰ_±_(**ξ**) = ħ^2^**k**^2^/2*m* ± ∣**d**(ξ)∣. The corresponding wave functions have nontrivial phase-space geometry encoded in the Berry curvatures

$$\Omega_{\alpha ,\beta }^{\pm }(\xi )=\pm \frac{1}{2}\hat{d}(\xi )\cdot (\partial_{\alpha }\hat{d}(\xi )\;\times \;\partial_{\beta }\hat{d}(\xi ))$$ (4)

each corresponding to one of the six orthogonal planes in the 4D phase space spanned by **ξ**. The band index is a constant of motion in the semiclassical theory, and each electronic band may be treated independently; we will suppress the band index unless necessary.

The curvatures modify the equations of motion and the invariant measure in phase space. To simplify the notation, we introduce a 4 × 4 matrix

$${\left[\Gamma (\xi )\right]}_{\alpha ,\beta }=\Omega_{\alpha ,\beta }(\xi )-{\left[i\sigma_{y}⊗1\right]}_{\alpha ,\beta }$$ (5)

to write the equations of motion

$${\overset{\cdot }{\xi }}_{\alpha }(\xi )={\left[\Gamma^{-1}(\xi )\right]}_{\alpha \beta }\;(\partial_{\beta }\tilde{E}(\xi )+\mathrm{eE}\;\delta_{\beta ,y})/\hbar$$ (6)

where *E* is the external electric field along the $\hat{y}$ direction, and the electron charge is (−*e*). Here, $\tilde{E}(\xi )≃E(\xi )$ up to corrections of order (λ/*E*_F_)(*a*/*L*_s_) that can be ignored in the regime of interest. Our compact notation hides all the familiar terms, including the anomalous velocity, inside Γ^−1^ (see Supplementary Text for more details).

The combination of a spatially varying magnetic texture and SOC leads to finite real-space, momentum-space, and mixed real-momentum–space curvatures. The electrons acquire an anomalous velocity proportional to the momentum-space Berry curvature Ω_*k_x_*,*k_y_*_, an “anomalous force” proportional to the real-space Berry curvature Ω_*x*,*y*_ and corrections to the group velocity, and generalized force proportional to the mixed real-momentum–space Berry curvatures.

Crucially, in addition to the equations of motion, the curvatures also modify the volume element that remains invariant under phase-space flows. Thus, to satisfy Liouville’s theorem, one must use the integration measure $dV_{\xi }=\sqrt{\text{det}\;[\Gamma (\xi )]}\;d^{4}\xi /{\left(2\pi \right)}^{2}V$, where *V* is the volume of the system (*13*). We note that in the presence of an external magnetic field $B_{z}\hat{z}$, $\sqrt{\text{det}\;[\Gamma (\xi )]}$ reduces to the well-known factor of (1 + *e*Ω_*k_x_*,*k_y_*_*B_z_*/ħ) when only the momentum-space curvature is present; however, we will need the more general result here.

### Hall conductivity

With electric field applied along $\hat{y}$, we must calculate the transverse current along $\hat{x}$

$$j_{x}=-e\int {\mathrm{dV}}_{\xi }\;\overset{\cdot }{x}(\xi )\;f(\xi )$$ (7)

where *f*(**ξ**) is the electronic distribution function. The distribution function reduces to the equilibrium Fermi-Dirac function *f*^0^[ℰ(**ξ**)] in the absence of the external electric field. The goal is to find contributions that are linear order in *E* to calculate the electric conductivity.

The anomalous Hall contribution to the current derives from the intrinsic anomalous velocity and couples to the equilibrium distribution function *f*^0^[ℰ(ξ)]. We isolate the terms in $\overset{\cdot }{x}$ linear in *E* to find

$$\sigma_{\mathrm{xy}}^{\text{AHE}}=-\frac{e^{2}}{\hbar }\sum_{l=\pm }\int \frac{d^{2}r\;d^{2}k}{{\left(2\pi \right)}^{2}V}\;\Omega_{k_{x},k_{y}}^{l}(\xi )\;f_{l}^{0}[E_{l}(\xi )]$$ (8)

where *l* = ± indexes the two bands. We emphasize that $\sqrt{\text{det}\;[\Gamma (\xi )]}$ in the measure exactly cancels the determinant factor in Γ^−1^(**ξ**) so that the final answer depends only on the momentum-space Berry curvature. We further expand Ω_*k_x_*,*k_y_*_(**ξ**) to the lowest order in λ/*J* to find

$$\sigma_{\mathrm{xy}}^{\text{AHE}}\approx -\frac{e^{2}a^{2}}{2\hbar }\;{\bar{m}}_{z}{\left(\lambda /J\right)}^{2}\sum_{l=\pm }\;l\;n_{l}$$ (9)

where ${\bar{m}}_{z}=\int d^{2}r\;{\hat{m}}_{z}(r)/V$ is the average out-of-plane magnetization and the band-resolved density is *n_l_* = ∫ *d*^2^**k** *f*^0^[ℰ*_l_*(**k**)]/(2π)^2^ with ℰ*_l_*(**k**) ***=***ℰ_***l***_(k; λ ***=*** **0**).

The corresponding resistivity is found from the conductivity via $\rho_{\mathrm{xy}}=-\sigma_{\mathrm{xy}}/(\sigma_{\mathrm{xx}}^{2}+\sigma_{\mathrm{xy}}^{2})$, where σ*_xy_* ≪ σ*_xx_* = (*e*^2^/*h*)*k_F_*𝓁. This relationship will be used to convert conductivities to resistivities for each contribution to the Hall effect. For the AHE, this leads to the scaling relation $\rho_{\mathrm{xy}}^{\text{AHE}}∼{\left(\lambda /E_{F}\right)}^{2}{\left(a/\ell \right)}^{2}$.

All other contributions to the Hall response involve the electric field–induced perturbations to the distribution function determined by solving the Boltzmann equation. We expand the distribution function to linear order in the electric field, *f* = *f*^0^ + *g* + O(*E*^2^), and substitute it into the Boltzmann equation with a relaxation time τ = 𝓁/*v*_F_ to find the equation for *g*

$$\left(1+\tau {\overset{\cdot }{\xi }}^{\left(I\right)}\cdot \nabla_{\xi })g(\xi )=-\tau \;{\overset{\cdot }{\xi }}^{\left(D\right)}\cdot \nabla_{\xi }f^{0}[E(\xi )\right]$$ (10)

where ${\overset{\cdot }{\xi }}^{\left(I\right)}$ and ${\overset{\cdot }{\xi }}^{\left(D\right)}$ are the electric field–independent and electric field–dependent parts of $\overset{\cdot }{\xi }$ in Eq. 6. We now take advantage of the fact that $\tau {\overset{\cdot }{\xi }}^{\left(I\right)}\cdot \nabla_{\xi }∼(\ell /L_{s})(a/L_{s})\ll 1$ when 𝓁/*L*_s_ ≪ 1 to invert the operator on the left-hand side and solve for *g*(**ξ**). This is analogous to the Zener-Jones calculation (*33*) of the Hall conductivity in the weak field regime ω_c_τ ≪ 1. Solving the Boltzmann equation for *L*_s_ ≪ 𝓁 is technically much harder. We will investigate aspects of this regime using the Kubo formalism below.

The term *g*^(1)^(**ξ**) linear in τ does not contribute to the Hall conductivity, and the leading order contribution proportional to τ^2^ is

$$g^{\left(2\right)}(\xi )=\tau^{2}\;{\overset{\cdot }{\xi }}^{\left(I\right)}\cdot \nabla_{\xi }({\overset{\cdot }{\xi }}^{\left(D\right)}\cdot \nabla_{\xi }f^{0}[E(\xi )])$$ (11)

We emphasize that this equation involves all six curvatures along with mixed derivatives of the semiclassical eigenenergies. Combining *g*^(2)^(**ξ**) with Eq. 7, we calculate the current that is linear in *E*

$$j_{x}^{\left(2\right)}=-e\tau^{2}\int {\mathrm{dV}}_{\xi }\;{\overset{\cdot }{x}}^{\left(I\right)}(\xi )\;{\overset{\cdot }{\xi }}^{\left(I\right)}\cdot \nabla_{\xi }({\overset{\cdot }{\xi }}^{\left(D\right)}\cdot \nabla_{\xi }f^{0}[E(\xi )])$$ (12)

We organize the calculation of the conductivity by classifying the various terms in Eq. 12 in powers of the small parameters λ/*E*_F_ and *a*/*L*_s_ (see Supplementary Text for details). We now discuss the leading order contributions.

We first focus on the zeroth-order term in (λ/*E*_F_). Without SOC, all curvatures vanish except the real-space curvature that leads to the topological Hall contribution

$${\sigma_{\mathrm{xy}}^{\text{THE}}=\frac{e^{2}\tau^{2}}{\hbar^{3}}\;n_{\text{sk}}\;\sum_{l=\pm }K_{l}(\mu )|}_{\lambda =0}$$ (13)

Here, $n_{\text{sk}}=\int d^{2}r\;\hat{m}\cdot (\partial_{x}\hat{m}\;\times \;\partial_{y}\hat{m})/(4\pi V)$ is the skyrmion density and

$$K_{\pm }(\mu )=\mp \hbar^{4}\int \frac{d^{2}k}{\left(4\pi \right)}(\frac{\partial f_{\pm }^{0}}{\partial E})v^{T}(M^{-1}-\text{Tr}M^{-1})v$$ (14)

is a Fermi surface integral that depends on the chemical potential μ (or filling *n*) and the band index. Here, **v** = **∇**_**k**_ℰ(**ξ**)/ħ is the band velocity vector, and $M_{\mu \nu }^{-1}=\partial_{k_{\mu },k_{\nu }}E(\xi )/\hbar^{2}$ is the inverse mass tensor. The semiclassical theory illuminates the relationship between the real-space Berry curvature that is a property of the spatial evolution of the semiclassical Bloch eigenstates and the skyrmion density that is a property of the spatial evolution of the magnetization vector. In the absence of SOC, $\Omega_{x,y}^{\pm }=\mp \hat{m}\cdot (\partial_{x}\hat{m}\;\times \;\partial_{y}\hat{m})/2$. The result of Eq. 13 bears a notable resemblance to the canonical solution (*33*) for the semiclassical Hall conductivity with the real-space Berry curvature Ω_*x*,*y*_ playing the role of an external magnetic field, in agreement with the intuitive picture behind THE. The corresponding resistivity is independent of τ and scales as $\rho_{\mathrm{xy}}^{\text{THE}}∼{\left(a/L_{s}\right)}^{2}$.

Next, we focus on terms linear in (λ/*E*_F_). Although there are several terms, there is only one that is linear in (*a*/*L*_s_). It originates from mixed spatial- and momentum-space derivatives of the semiclassical energies ℰ(**ξ**) and is independent of all Berry curvatures

$${\delta \sigma }_{\mathrm{xy}}=-\frac{e^{2}\tau^{2}}{2m}\sum_{l=\pm }\;\omega_{l}\;n_{l}$$ (15)

where $\omega_{l}=1/V\int d^{2}r\;\hat{z}\cdot (\nabla_{r}\;\times \;v_{l}(r))$ is the average “vorticity” of electrons in band *l* with velocity **v**_**l**_(**r**) that is linear in λ (see Supplementary Text for details) and *n_l_* is the band-resolved density defined below Eq. 9. The intuition behind this term is that real-space gradients of the magnetic texture can lead to orbital electronic motion akin to the dynamics induced by an external magnetic field. For the Rashba SOC considered here, the vorticity simplifies to $∼\int dr\;\nabla_{r}\cdot \hat{m}(r)$. This term has been discussed in the literature (*24*, *26*, *29*, *30*) as a O(λ) correction to the emergent magnetic field arising from skyrmions. Here, this contribution arises not from SOC corrections to the real-space Berry curvature but instead from mixed momentum- and real-space derivatives of the semiclassical eigenvalues. Similar to THE, the corresponding resistivity is independent of τ but instead scales as δρ*_xy_* ∼ (*a*/*L*_s_)(λ/*E*_F_). We note, however, that δρ*_xy_* vanishes identically for any periodic spin texture, such as a skyrmion crystal. More generally, for any smooth texture for which **v**(**r**) has continuous first-order partial derivatives, we can use Stokes’ theorem and show that the vorticity leads only to a boundary term that is negligible in the thermodynamic limit.

All other contributions to σ*_xy_*, including the mixed curvature terms, scale as (λ/*J*)^2^ (𝓁/*L*_s_)^2^ and hence will be negligible compared to the dominant anomalous Hall $\sigma_{\mathrm{xy}}^{\text{AHE}}∼{\left(\lambda /J\right)}^{2}$ and topological Hall $\sigma_{\mathrm{xy}}^{\text{THE}}∼{\left(\ell /L_{s}\right)}^{2}$ contributions (see Supplementary Text for details). Thus, we have used the semiclassical approach that treats all curvatures on equal footing to conclude that AHE and THE are additive and the largest contribution to the Hall effect for *L*_s_ ≫ 𝓁.

We briefly comment on (*37*), which presents a Hall calculation that ignores the spatial dependence of the distribution function and focuses only on the mixed curvature, neglecting Ω_*k_x_*,*k_y_*_ and Ω_*x*,*y*_. Thus, they do not obtain our result (Eq. 1), which is just the sum of THE and the intrinsic AHE.

### Kubo formula analysis

We next turn to the opposite limit of small skyrmions such that $a\approx k_{F}^{-1}≲L_{s}\ll \ell$ and numerically demonstrate that, although the regime of interest is physically very different from the semiclassical regime described above, some of the qualitative features of the Hall signal remain intact. We set the mean-free path to infinity and use an exact Kubo formula to numerically calculate the Hall conductivity for a lattice model of itinerant electrons in the presence of a skyrmion crystal (see Supplementary Text for details). The starting Hamiltonian is a tight-binding generalization of Eq. 2 describing electrons on a lattice with nearest neighbor hopping *t* and Rashba SOC λ, coupled to a background spin texture described by local moments **m**_**i**_ at each lattice site **i**. The skyrmion crystal defines an enlarged *N*_s_ × *N*_s_ unit cell, where *N*_s_ = *L*_s_/*a*, and results in a magnetic Brillouin zone with $N_{b}=2N_{s}^{2}$ bands. We present here results for a triangle lattice, but as we show in Supplementary Text, our results are independent of the lattice for low densities.

We use exact diagonalization to compute the energy eigenvalues and eigenfunctions of our lattice Hamiltonian and then use the TKNN formula (*35*) to determine the Hall conductivity in terms of the momentum-space Berry curvature in the magnetic Brillouin zone. Note that this numerically exact procedure includes all the effects of the anomalous velocity and the real-space Berry curvature arising from the skyrmions; however, unlike the semiclassical theory, it is hard to decompose the final result into AHE and THE contributions. We thus proceed as follows. We first show that in various limits, one obtains just the AHE (in a ferromagnetic background) or just the THE (in a skyrmion crystal with λ = 0). Last, we consider the full problem and gain qualitative insights into the numerical results by comparing them with the semiclassical results described above.

First, consider the simplest ferromagnetic case with uniform magnetization ${\hat{m}}_{i}=\hat{z}$ (independent of **i**). which is just the lattice version of the continuum model analyzed in (*13*) with their Δσ*_z_* corresponding to our *J*σ*_z_*. An AHE is seen in this case, provided that both λ and *J* are nonzero. The SOC λ breaks the twofold spin degeneracy of the bands everywhere except at the time-reversal invariant momenta (TRIM) where time reversal (TR) enforces a Kramers degeneracy. A nonzero *J* destroys TR symmetry, causes band inversion, and creates Berry curvature hotspots at TRIMs that then lead to an enhancement of the AHE conductivity whenever the Fermi level falls near the TRIM points.

**Fig. 2.**
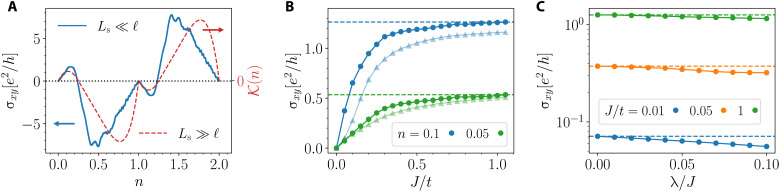
Kubo formula results for Hall conductivity. (**A**) The blue curve is THE calculated at λ = 0 with *J*/*t* = 10. The red curve shows $K=K_{+}+K_{-}$ (Eq. 14) plotted as a function of density *n*, which describes how the band structure controls the semiclassical result of Eq. 13. Despite their different regimes of validity, the Kubo σ*_xy_* and semiclassical K(n) have the same sign and vanish at the van Hove fillings where the Fermi surface undergoes a Lifshitz transition. (**B**) σ*_xy_* as a function of *J*/*t* for two densities *n* = 0.1 (blue) and 0.05 (green); circles are at λ = 0 and triangles at λ/*t* = 0.1. σ*_xy_* rises linearly for small *J*/*t* and asymptotes at large *J*/*t* to a constant that scales roughly with *n*, consistent with the semiclassical results (see the main text). The *n* dependence of the slope at small *J*/*t* is not seen in the semiclassical regime. (**C**) Variation of σ*_xy_* (log scale) with λ/*J* at fixed *n* = 0.1 for different *J*/*t* values. The fractional change due to SOC is seen to be largest at small *J*/*t*.

We next look at a skyrmion crystal but set λ = 0 so that there is no AHE (although the net *M_z_* is nonzero). The Fourier modes of the periodic texture cause scattering between momentum eigenstates and lead to band folding. At strong coupling *J*/*t* ≫ 1, the bands separate into two sectors with the spins aligned/antialigned with the local magnetic texture. The corresponding Hall conductivity is the THE arising from a nonzero skyrmion number. It shows a nontrivial dependence on the band filling as seen in Fig. 2A (blue curve). Comparing this with the semiclassical THE prediction of Eq. 13 (red curve), we see that these results, although obtained in very different regimes, share some qualitative features. Both have the same sign at each density and vanish at the van Hove filling where the Fermi surface undergoes a Lifshitz transition.

Next, we examine the *J*/*t* dependence of the Kubo results for σ*_xy_* in Fig. 2B. We see a linear regime at small *J*/*t* crossing over to saturation at large *J*/*t*. We can gain some insight into these results, at least at λ = 0, by looking at the *J* dependence of the semiclassical result (Eq. 13), which predicts both an initial linear rise and an asymptotic large *J* value that scales with *n* (see Supplementary Text). However, the semiclassical result has an initial slope independent of density *n*, unlike what is seen in Fig. 2B.

Last, we turn to the SOC dependence of the Kubo results. From Fig. 2B, we already see that the relative effect of λ is smaller in the “strong coupling” regime *J*/*t* ≳ 1. To see this more clearly, we plot in Fig. 2C σ*_xy_* on a log scale as a function of λ/*J* for various values of *J*/*t*. We see that the effect of SOC is, in general, small, with the largest fractional change observed for small *J*/*t*. The λ-dependent change to the topological Hall signal at λ = 0 is, by definition, some form of an AHE. The numerically exact Kubo results, however, do not allow us to definitively identify its physical origin, which could be related to, e.g., the intrinsic AHE proportional to the magnetization *M_z_* or the chiral Hall effect arising from a single spatial gradient of the magnetic texture (*31*).

## DISCUSSION

We have presented a complete semiclassical analysis in the weak SOC λ ≪ *E*_F_ regime for *a* ≪ 𝓁 ≪ *L*_s_ and demonstrated that the Hall resistivity is the sum of an anomalous Hall contribution, arising from the momentum-space Berry curvature and proportional to the average out-of-plane magnetization, and a topological Hall contribution, arising from the real-space Berry curvature and proportional to the skyrmion density. All corrections were explicitly shown to be higher order in the small parameters. The semiclassical results are valid for any spin texture without any assumption about its periodicity. In the opposite limit *L*_s_ ≪ 𝓁 = ∞ (zero disorder), we have presented exact Kubo formula results for skyrmion crystals.

We conclude by noting effects that we have not included and questions for further study. We focused on the intrinsic AHE, arising for momentum-space Berry curvature, often the dominant contribution (*12*) to the AHE, but did not consider extrinsic effects such as skew and side jump scattering. We have also not analyzed nonperiodic spin textures that vary on a length scale *L*_s_ ≲ 𝓁 (*23*). Such a regime has been analyzed (*32*) in the context of electrons scattering off a single skyrmion with the prediction of a noncollinear Hall effect proportional to the SOC. It would be interesting to extend our semiclassical analysis to this regime.

In the semiclassical regime that we have examined in detail, we found a previously unidentified vorticity term that is linear in λ (Eq. 15). We were able to use Stokes’ theorem to reduce it to a boundary term that vanishes for periodic textures. An interesting question for further study is the fate of this term in the presence of singularities, such as Bloch points, that may act as obstructions to the use of Stokes theorem.

## Supplementary Materials

### This PDF file includes:

Semiclassical equations of motion with phase-space Berry curvatures Solution to the Boltzmann equation Hall conductivity independent of curvatures Semiclassical theory for general dispersions and SOCs Kubo formula calculation Figs. S1 and S2 References
