# Supplementary material for: Unified theory of the anomalous and topological Hall effects with phase-space Berry curvatures
Source: Sci Adv. 2022 Nov 9;8(45):eabq2765. doi: 10.1126/sciadv.abq2765 (PMC9645717; doi:10.1126/sciadv.abq2765)
Supplement: Supplementary file 1 — Semiclassical equations of motion with phase-space Berry curvatures Solution to the Boltzmann equation Hall conductivity independent of curvatures Semiclassical theory for general dispersions and SOCs Kubo formula calculation Figs. S1 and S2 References [file sciadv.abq2765_sm.pdf]

Science Advances
AAAS

Supplementary Materials for

Unified theory of the anomalous and topological Hall effects with phase-space Berry curvatures

Nishchhal Verma *et al.*

Corresponding author: Mohit Randeria, randeria.1@osu.edu

Sci. Adv. **8**, eabq2765 (2022)
DOI: 10.1126/sciadv.abq2765

This PDF file includes:

Semiclassical equations of motion with phase-space Berry curvatures
Solution to the Boltzmann equation
Hall conductivity independent of curvatures
Semiclassical theory for general dispersions and SOCs
Kubo formula calculation
Figs. S1 and S2
References

Semiclassical equations of motion with phase space Berry curvatures

Semiclassical theory describes transport in terms of electron wave-packets whose width is larger than microscopic lattice scale a but much smaller than mean-free path ℓ so that the average position $\mathbf{r}$ and average momentum $\mathbf{k}$ of the wavepacket are well-defined simultaneously. This is in addition to their time evolution which is governed by a semiclassical Hamiltonian. The magnetic texture presents a new length-scale related to its size L_s. The construction now requires a gradient expansion which introduces an additional constraint that the width is smaller than L_s. The Hamiltonian thus obtained is a function of phase-space variables $\boldsymbol{\xi} = (x, y, k_x, k_y)$:

$$\mathcal{H}(\boldsymbol{\xi}) = \frac{\hbar^2\mathbf{k}^2}{2m} + \mathbf{d}(\boldsymbol{\xi})\cdot\boldsymbol{\sigma}, \quad \mathbf{d}(\boldsymbol{\xi}) = a\lambda(\mathbf{k}\times\hat{\mathbf{z}}) - J\hat{\mathbf{m}}(\mathbf{r}), \quad \mathcal{E}_\pm(\boldsymbol{\xi}) = \frac{\hbar^2\mathbf{k}^2}{2m} \pm |\mathbf{d}(\boldsymbol{\xi})| \tag{1}$$

and hosts six types of Berry curvatures, each corresponding to a plane in the 4D phase space

$$\Omega^\pm_{\alpha,\beta}(\boldsymbol{\xi}) \;=\; \pm\frac{1}{2}\hat{\mathbf{d}}(\boldsymbol{\xi})\cdot\left(\partial_\alpha\hat{\mathbf{d}}(\boldsymbol{\xi})\times\partial_\beta\hat{\mathbf{d}}(\boldsymbol{\xi})\right) \tag{2}$$

where $\pm$ label the two bands. The curvatures introduce non-trivial Poisson bracket relations between the phase space variables that lead to corrections in the equations of motion and the invariant measure. Both these quantities are captured by the completely anti-symmetric matrix

$[\Gamma(\boldsymbol{\xi})]_{\alpha,\beta} = \Omega_{\alpha,\beta}(\boldsymbol{\xi}) - [i\sigma_y \otimes \mathbb{1}]_{\alpha,\beta}$ as defined in the main text. Here we explicitly write the expression for completeness:

$$\hbar \begin{pmatrix} \dot{x} \\ \dot{y} \\ \dot{k}_x \\ \dot{k}_y \end{pmatrix} = \left[\begin{pmatrix} 0 & \Omega_{k_x,k_y} & -\Omega_{y,k_y} & \Omega_{y,k_x} \\ -\Omega_{k_x,k_y} & 0 & \Omega_{x,k_y} & -\Omega_{x,k_x} \\ \Omega_{y,k_y} & -\Omega_{x,k_y} & 0 & \Omega_{x,y} \\ -\Omega_{y,k_x} & -\Omega_{x,k_x} & -\Omega_{x,y} & 0 \end{pmatrix} - \begin{pmatrix} 0 & 0 & -1 & 0 \\ 0 & 0 & 0 & -1 \\ 1 & 0 & 0 & 0 \\ 0 & 1 & 0 & 0 \end{pmatrix}\right] \times \frac{1}{\sqrt{\det[\Gamma(\boldsymbol{\xi})]}} \begin{pmatrix} \partial_x \widetilde{\mathcal{E}}(\boldsymbol{\xi}) \\ \partial_y \widetilde{\mathcal{E}}(\boldsymbol{\xi}) + eE \\ \partial_{k_x} \widetilde{\mathcal{E}}(\boldsymbol{\xi}) \\ \partial_{k_y} \widetilde{\mathcal{E}}(\boldsymbol{\xi}) \end{pmatrix} \tag{3}$$

$$dV_{\boldsymbol{\xi}} = \frac{d^2\mathbf{r} d^2\mathbf{k}}{(2\pi)^2 V} \sqrt{\det[\Gamma(\boldsymbol{\xi})]} \tag{4}$$

There is one additional change in the equations. The non-trivial spatial and momentum variation in the eigenfunction of the semiclassical Bloch Hamiltonian $|u(\boldsymbol{\xi})\rangle$ leads to a shift in the energy $\widetilde{\mathcal{E}}(\boldsymbol{\xi}) = \mathcal{E}(\boldsymbol{\xi}) + \delta\mathcal{E}(\boldsymbol{\xi})$ with

$$\delta\mathcal{E}(\boldsymbol{\xi}) = -\sum_{i=x,y} \mathrm{Im}\left[\Big(\partial_{r_i}\langle u(\boldsymbol{\xi})|\Big)(\mathcal{E}(\boldsymbol{\xi}) - \mathcal{H}(\boldsymbol{\xi}))\Big(\partial_{k_i}|u(\boldsymbol{\xi})\rangle\Big)\right]. \tag{5}$$

We can ignore $\delta\mathcal{E}(\boldsymbol{\xi})$ in our calculation because it scales as $(\lambda/E_F)(a/L_s)$ and thus leads to higher order corrections to the Hall effect not considered here.

The matrix representation in eq. (3) contains all contributions of the curvatures. In particular, the anomalous velocity can be extracted from the electric field dependent part of the velocity in $+x$ direction

$$\dot{x}^{(D)}(\boldsymbol{\xi}) = \frac{e}{\hbar} \frac{\Omega_{k_x,k_y}(\boldsymbol{\xi})}{\sqrt{\det[\Gamma(\boldsymbol{\xi})]}} E \tag{6}$$

The determinant factor in the denominator may seem unfamiliar but is absolutely crucial for calculating the correct intrinsic anomalous Hall response. There is a complete cancellation of the phase-space measure factors in the Hall current so that the anomalous Hall conductivity

only depends on the momentum-space Berry curvature:

$$j_x = e \int \frac{d^2\mathbf{r} d^2\mathbf{k}}{(2\pi)^2 V} \sqrt{\det[\Gamma(\boldsymbol{\xi})]}\, \dot{x}^{(D)}(\boldsymbol{\xi})\ f^0[\mathcal{E}_l(\boldsymbol{\xi})] = \left(\frac{e^2}{\hbar} \int \frac{d^2\mathbf{r} d^2\mathbf{k}}{(2\pi)^2 V}\ \Omega_{k_x,k_y}(\boldsymbol{\xi})\ f^0[\mathcal{E}_l(\boldsymbol{\xi})] \right) E \tag{7}$$

The quantity within brackets is $\sigma_{xy}^{\mathrm{AHE}}$. Even though the expression contains only the momentum-space Berry curvature $\Omega_{k_x,k_y}(\boldsymbol{\xi})$, we must keep in mind that $\Omega_{k_x,k_y}(\boldsymbol{\xi})$ is a function of momentum and real space and the two integrals are not separable:

$$\sigma_{xy}^{\mathrm{AHE}} = -\sum_{l=\pm} l \frac{e^2}{\hbar} \int \frac{d^2\mathbf{r}\ d^2\mathbf{k}}{(2\pi)^2 V} \left[\frac{a^2 \lambda^2 J m_z(\mathbf{r})}{2|\mathbf{d}(\boldsymbol{\xi})|^3} \right] f^0[\mathcal{E}_l(\boldsymbol{\xi})] \tag{8}$$

Since there is already an explicit λ^2 and λ/E_F is a small parameter, we can set $\lambda = 0$ in the rest of the expression to find the leading contribution. The spatial dependence in the semiclassical eigenenergies drops out when $\lambda = 0$, that is $(\mathcal{E}_l(\boldsymbol{\xi}; \lambda = 0) = \mathcal{E}_l(\mathbf{k}))$ and the spatial and momentum integrals become separable

$$\sigma_{xy}^{\mathrm{AHE}} \approx -\sum_{l=\pm} l \frac{e^2 a^2}{2\hbar} \left(\frac{\lambda}{J} \right)^2 \left(\int \frac{d^2\mathbf{r}}{V}\ m_z(\mathbf{r}) \right) \left(\int \frac{d^2\mathbf{k}}{(2\pi)^2} f^0[\mathcal{E}_l(\mathbf{k})] \right) \tag{9}$$

We thus find that the intrinsic contribution only probes the net out-of-plane magnetization even for spatially varying textures.

Solution to the Boltzmann equation

Focussing on contributions that come from electric field induced perturbations to the distribution function, we write the full distribution function in the presence of electric field as $f = f^0 + g$ where g is linear order in the field. We then use the relaxation time approximation to write the Boltzmann equation as

$$\dot{\boldsymbol{\xi}} \cdot \boldsymbol{\nabla}_{\boldsymbol{\xi}} \left(f^0(\boldsymbol{\xi}) + g(\boldsymbol{\xi}) \right) = -\frac{g(\boldsymbol{\xi})}{\tau} \tag{10}$$

We write $\dot{\boldsymbol{\xi}} = \dot{\boldsymbol{\xi}}^{(I)} + \dot{\boldsymbol{\xi}}^{(D)}$ where I and D refer to electric field dependent and independent components to find an equation for $g(\boldsymbol{\xi})$

$$\left(1 + \tau\dot{\boldsymbol{\xi}}^{(I)} \cdot \boldsymbol{\nabla}_{\boldsymbol{\xi}}\right) g(\boldsymbol{\xi}) = -\tau\, \dot{\boldsymbol{\xi}}^{(D)} \cdot \boldsymbol{\nabla}_{\boldsymbol{\xi}} f^0[\mathcal{E}(\boldsymbol{\xi})] \tag{11}$$

The differential operator on the left has a particular scaling. With $g(\boldsymbol{\xi}) \sim g[\mathcal{E}(\boldsymbol{\xi})]$, we can infer that $\tau\dot{\boldsymbol{\xi}}^{(I)} \cdot \boldsymbol{\nabla}_{\boldsymbol{\xi}}\mathcal{E} \sim (\ell/L_s)(a/L_s)$. Now since $a \ll \ell \ll L_s$, both these ratios are small and hence, we can invert the operator to find

$$g(\boldsymbol{\xi}) = -\tau\, \left(1 - \tau\dot{\boldsymbol{\xi}}^{(I)} \cdot \boldsymbol{\nabla}_{\boldsymbol{\xi}}\right)\, \dot{\boldsymbol{\xi}}^{(D)} \cdot \boldsymbol{\nabla}_{\boldsymbol{\xi}} f^0[\mathcal{E}(\boldsymbol{\xi})] = g^{(1)}(\boldsymbol{\xi}) + g^{(2)}(\boldsymbol{\xi}) \tag{12}$$

where the superscripts label the order in τ. The first order term $g^{(1)}(\boldsymbol{\xi})$ does not result in any Hall conductivity. While we find that at the end of a long calculation, we can use time-reversal (TR) symmetry to understand why it vanishes. Onsager's reciprocity relation forces Hall conductivity to be odd under TR. The conductivity arising from $g^{(1)}(\boldsymbol{\xi})$ doesn't have this property:

$$\sigma_{xy} \sim \frac{e}{V}\int \frac{d^2\mathbf{r}d^2\mathbf{k}}{(2\pi)^2}\, \underbrace{\sqrt{\det[\Gamma(\boldsymbol{\xi})]}}_{\text{TR even}}\, \underbrace{\dot{x}(\boldsymbol{\xi})}_{\text{TR odd}}\, \Big(-\tau\, \underbrace{\dot{\boldsymbol{\xi}}^{(D)}}_{\text{TR odd}} \cdot \underbrace{\boldsymbol{\nabla}_{\boldsymbol{\xi}} f^0[\mathcal{E}(\boldsymbol{\xi})]}_{\text{TR even}}\Big) \tag{13}$$

It is clearly even under time-reversal and hence must vanish. The contributions from $g^{(2)}(\boldsymbol{\xi})$ survive this argument.

The calculation for Hall conductivity involves combining the distribution function with velocity and the appropriate phase space volume factor. The algebra is tedious but there are a few simplifying factors. Products and derivatives of the curvatures can be excluded as they are all higher order in (a/L_s). There will still be many terms and hence we need to introduce a classification scheme for bookkeeping

$$\prod_{i=1}^{m}\prod_{j=1}^{n} \partial_{k_i}\partial_{r_j}(\cdot) \longrightarrow (m, n) \tag{14}$$

Here (m, n) labels expressions that have m momentum derivatives and n spatial derivatives. These numbers count both: derivatives in semiclassical energies and the implicit derivatives hidden inside the curvatures.

With these labels, the Hall conductivity is

$$\sigma_{xy} \sim \int \frac{d^2\mathbf{r}d^2\mathbf{k}}{(2\pi)^2 V} \underbrace{\text{Phase space volume}}_{(0,0)+(1,1)} \times \underbrace{\text{Velocity}}_{(1,0)+(2,1)} \times \underbrace{\dot{\boldsymbol{\xi}}^{(I)} \cdot \boldsymbol{\nabla}_{\boldsymbol{\xi}}}_{(1,1)+(2,2)} \times \underbrace{\left(\dot{\boldsymbol{\xi}}^{(D)} \cdot \boldsymbol{\nabla}_{\boldsymbol{\xi}} f^0[\mathcal{E}(\boldsymbol{\xi})]\right)}_{(1,0)+(2,1)} \tag{15}$$

Each individual piece has two tuples. The first tuple labels contributions from semiclassical eigenenergies and the second labels contributions from the Berry curvatures. For instance, in phase-space volume, $(0,0)$ labels the constant and $(1,1)$ labels $\Omega_{k_x,x}$ and $\Omega_{k_x,y}$. If we further focus on contributions to the full Hall response that have at most one curvature, there are only two broad categories: no curvature $(3,1)$ and one curvature $(4,2)$. The number of terms inside each category is still quite large.

We turn to energy scaling relations and take advantage of the fact that λ/E_F is a small parameter. To the leading order, we find that $\partial_{k_x}\mathcal{E} \sim \lambda^0$, $\Omega_{x,y} \sim \lambda^0$, $\partial_x\mathcal{E} \sim \lambda$, , $\Omega_{x,k_y} \sim \lambda$ and $\Omega_{k_x,k_y} \sim \lambda^2$. We are now ready to calculate the contributions order by order in λ/E_F and a/L_s:

- Zeroth order in λ/E_F

  The equations of motion are quite simple since all spatial derivatives vanish. There are no $(3,1)$ terms and the only non-zero $(4,2)$ term has both spatial derivatives coming from the real-space Berry curvature. The resulting contribution is the Topological Hall response σ_{xy}^{THE}. Simplifying eq. 13 we find

$$\sigma_{xy}^{\text{THE}} = -\frac{2e^2\tau^2}{m\hbar} n_{\text{sk}} \begin{cases} \pi\hbar^2 n/m, & \text{for } J > \pi\hbar^2 n/m \\ J, & \text{for } J < \pi\hbar^2 n/m \end{cases} \tag{16}$$

  where n is the electron density. Hence as J/E_F is tuned, σ_{xy} crosses over from a linear in J regime to a saturating value that is independent of J but increases with density. Here n_{sk} is the skyrmion density

$$n_{\text{sk}} = \frac{1}{V} \int \frac{d^2\mathbf{r}}{4\pi}\ \hat{\mathbf{m}}(\mathbf{r}) \cdot (\partial_{\mathbf{x}}\hat{\mathbf{m}}(\mathbf{r}) \times \partial_{\mathbf{y}}\hat{\mathbf{m}}(\mathbf{r})) \tag{17}$$

For small densities $n < \dfrac{mJ}{\pi\hbar^2}$ this can be written as

$$\sigma_{xy}^{\text{THE}} = \frac{ne^2\tau}{m}\left(\frac{\tau e B_{\text{eff}}}{m}\right) \tag{18}$$

with $eB_{\text{eff}} = -2\pi n_{\text{sk}}\hbar$ acting like an effective magnetic field induced by the pressence of the spatially dispersive magnetic texture.

- First order in λ/E_F

There are both $(3,1)$ and $(4,2)$ type of contributions. We leave $(3,1)$ to the next section since it has a rather interesting origin, and focus on $(4,2)$, which has two possible origins.

The first involves $\Omega_{x,y}$ multiplied with four momentum derivatives of energies. We will now show that the resultant Hall conductivity is even in λ and hence is either zeroth order (discussed above) or second order (can be ignored). It can be checked that the semiclassical eigenenergies and the real-space curvature satisfy the relations

$$\mathcal{E}(\mathbf{r},\mathbf{k},\lambda) = \mathcal{E}(\mathbf{r},-\mathbf{k},-\lambda); \quad \Omega_{x,y}(\mathbf{r},\mathbf{k},\lambda) = \Omega_{x,y}(\mathbf{r},-\mathbf{k},-\lambda). \tag{19}$$

As a result, the integrand in phase space will switch λ upon flipping the momentum $\mathbf{k} \to -\mathbf{k}$. The resulting Hall conductivity changes $\sigma(\lambda) \to \sigma(-\lambda)$ under the flip. However, since $\mathbf{k}$ is a dummy variable that is being integrated over, Hall conductivity must satisfy $\sigma(\lambda) = \sigma(-\lambda)$ and is hence even in λ. There cannot be any first order corrections.

The other possibility both involves mixed curvatures, which as we showed, are at least linear in λ/E_F. Since the overall type has to be $(4,2)$, the pre-factors that come with mixed curvature should be of the type $(3,1)$. That is, there will be an additional spatial derivative in the full expression. It can either come from a different mixed curvature piece or from a first order spatial derivative of the semiclassical eigenenergies. It is easy to see that both these situations lead to second order contributions.

In sum, the only linear order contribution in SOC is of type $(3, 1)$. It is the subject of the next section.

Hall conductivity independent of curvatures

There are many simplifications when the curvatures are absent. We therefore find it instructive to present the full derivation, starting from the fact that the semiclassical energy is a function of both real space and momentum, $\mathcal{E}(\mathbf{r}, \mathbf{k})$. The derivation also appeals to the generality of the result and that it may apply to systems beyond the model Hamiltonian that we have considered in this paper.

With external Electric field, $\mathbf{E}$, the dynamics of the wave-packet is governed by the equations:

$$\begin{pmatrix} \dot{\mathbf{r}} \\ \dot{\mathbf{k}} \end{pmatrix} = \begin{pmatrix} 0 & \mathbb{1} \\ -\mathbb{1} & 0 \end{pmatrix} \begin{pmatrix} \boldsymbol{\nabla}_r \mathcal{E}/\hbar + e\mathbf{E}/\hbar \\ \boldsymbol{\nabla}_k \mathcal{E}/\hbar \end{pmatrix} \tag{20}$$

that lead to the following second-order shift in the distribution function

$$g^{(2)}(\boldsymbol{\xi}) = -\frac{e\tau^2}{\hbar^2} \left(\frac{\partial f^0}{\partial \mathcal{E}} \right) \big[\boldsymbol{\nabla}_k \mathcal{E} \cdot \boldsymbol{\nabla}_r - \boldsymbol{\nabla}_r \mathcal{E} \cdot \boldsymbol{\nabla}_k \big] \mathbf{E} \cdot \boldsymbol{\nabla}_k \mathcal{E} \tag{21}$$

and a Hall conductivity

$$\sigma_{\alpha\beta} = \frac{e^2\tau^2}{\hbar^3} \frac{1}{V} \int \frac{d^2\mathbf{r}\ d^2\mathbf{q}}{(2\pi)^2} \left(\frac{\partial f^0}{\partial \mathcal{E}} \right) (\partial_{k_\alpha} \mathcal{E}) \big[\boldsymbol{\nabla}_k \mathcal{E} \cdot \boldsymbol{\nabla}_r - \boldsymbol{\nabla}_r \mathcal{E} \cdot \boldsymbol{\nabla}_k \big] (\partial_{k_\beta} \mathcal{E}) \tag{22}$$

where we have suppressed the sum over the band index for brevity.

It is not obvious from the expression, as it stands, to see that the anti-symmetric response, $\sigma_{xy} - \sigma_{yx}$, is finite. Therefore, we next use integration by parts to rewrite the tensor as

$$\sigma_{\alpha\beta} = -\frac{e^2\tau^2}{\hbar^3} \frac{1}{V} \int d\mathbf{r}\ d\mathbf{q}\ f^0[\mathcal{E}]\ \big[\boldsymbol{\nabla}_k \partial_{k_\alpha} \mathcal{E} \cdot \boldsymbol{\nabla}_r - \boldsymbol{\nabla}_r \partial_{k_\alpha} \mathcal{E} \cdot \boldsymbol{\nabla}_k \big] (\partial_{k_\beta} \mathcal{E}) + \mathcal{S}_{\alpha\beta} \tag{23}$$

where $\mathcal{S}$ is a symmetric tensor, $\mathcal{S}_{\alpha\beta} = \mathcal{S}_{\beta\alpha}$, and the integrand is explicitly anti-symmetric

$$\big[\boldsymbol{\nabla}_k \partial_{k_\alpha} \mathcal{E} \cdot \boldsymbol{\nabla}_r - \boldsymbol{\nabla}_r \partial_{k_\alpha} \mathcal{E} \cdot \boldsymbol{\nabla}_k \big] (\partial_{k_\beta} \mathcal{E}) = \boldsymbol{\nabla}_k \partial_{k_\alpha} \mathcal{E} \cdot \boldsymbol{\nabla}_r \partial_{k_\beta} \mathcal{E} - \boldsymbol{\nabla}_r \partial_{k_\alpha} \mathcal{E} \cdot \boldsymbol{\nabla}_k \partial_{k_\beta} \mathcal{E} \tag{24}$$

Thus, the net anti-symmetric part can survive.

Back to our model Hamiltonian, we see that this effect cannot be described as an anomalous or topological Hall response. It survives in the absence of both Berry curvatures. As we will show now, its origin lies in vorticity of the local electronic velocity field. We expand the integrand

$$\partial^2_{k_x}\mathcal{E}\partial_{x,k_y}\mathcal{E} + \partial_{k_x,k_y}\mathcal{E}\partial_{y,k_y}\mathcal{E} - \partial_{x,k_x}\mathcal{E}\partial_{k_x,k_y}\mathcal{E} - \partial_{y,k_x}\mathcal{E}\partial^2_{k_y}\mathcal{E} \tag{25}$$

and use the fact that $\partial_{k_\alpha,k_\beta}\mathcal{E} = \delta_{\alpha,\beta}\ \hbar^2/m + \mathcal{O}(\lambda^2)$ to ignore the middle two terms when λ/E_F is small. The other two terms to first order can be written as

$$\frac{\hbar^2}{m}\left(\partial_{x,k_y}\mathcal{E} - \partial_{y,k_x}\mathcal{E}\right) = \frac{\hbar^2}{m}\left(\hat{\mathbf{z}}\cdot\nabla_{\mathbf{r}}\times(\nabla_{\mathbf{k}}\mathcal{E})\right) = \frac{\hbar^3}{m}\left(\hat{\mathbf{z}}\cdot\nabla_{\mathbf{r}}\times\mathbf{v}(\mathbf{r})\right) \tag{26}$$

An intuitive picture behind ordinary Hall effect is that electrons undertake cyclotron orbits under the action of the magnetic field. This results in electron velocity field forming vortices. This contribution, on the other hand, doesn't require an external magnetic field and instead uses the underlying magnetic texture to mimic vortices. The explicit connection to the texture is

$$\partial_{x,k_y}\mathcal{E}_\pm - \partial_{y,k_x}\mathcal{E}_\pm = \mp a\lambda\left(\partial_x m_x + \partial_y m_y\right) = \mp a\lambda\nabla\cdot\hat{m}(\mathbf{r}) \tag{27}$$

which has been reported elsewhere in the literature (26, 29, 30, 34) as a correction to the effective magnetic field in the presence of SOC. The resulting Hall conductivity for small densities $n < mJ/\pi\hbar$ is

$$\delta\sigma_{xy} = -\frac{ne^2\tau}{m}\left(\frac{\tau\lambda a}{\hbar}\int\frac{d^2\mathbf{r}}{V}\ \frac{\nabla_r\cdot\hat{\mathbf{m}}(\mathbf{r})}{2}\right) \tag{28}$$

and can be interpreted as arrising from an effective magnetic field $\sim \lambda\nabla_r\cdot\hat{\mathbf{m}}(\mathbf{r})$. Lastly, we note that this integral is a boundary term. Therefore unless there are singular features in the semiclassical velocity, the integral has to vanish. That being said the general result in eq. (22) may still be finite for systems with alternative kinetic dispersion relations $\mathcal{E}(\boldsymbol{\xi})$.

Semiclassical Theory for General Dispersions and Spin-orbit Couplings

Here we show that the main conclusions of the paper are unchanged by considering an electronic dispersion that is more general than the parabolic one considered in the main text or by considering a more general spin-orbit coupling. The semiclassical Bloch Hamiltonian for a general theory of electrons on a lattice in the presence of spin-orbit coupling and a local magnetic texture can be written as

$$\mathcal{H}(\boldsymbol{\xi}) = t(\boldsymbol{k})\mathbb{1} + \lambda\boldsymbol{\gamma}(\boldsymbol{k})\cdot\boldsymbol{\sigma} - J\hat{\boldsymbol{m}}(\boldsymbol{r})\cdot\boldsymbol{\sigma} \tag{29}$$

where the vector $\boldsymbol{\gamma}(\boldsymbol{k})$ describes an arbitrary spin-orbit coupling and $t(\boldsymbol{k})$ describes the electronic band structure in the absence of spin-orbit coupling. The semiclassical eigenvalues are

$$\mathcal{E}_{\pm}(\boldsymbol{\xi}) = t(\boldsymbol{k}) \pm |\lambda\boldsymbol{\gamma}(\boldsymbol{k}) - J\hat{\boldsymbol{m}}(\boldsymbol{r})| \tag{30}$$

The scaling of the real-space, momentum-space, and mixed real-momentum space Berry curvatures remain intact such that the leading order contributions to the Hall conductivity are still an anomalous and topological contribution. Using equation (8) the leading order anomalous Hall contribution can be written as

$$\sigma_{xy}^{\mathrm{AHE}} \approx -\frac{\lambda^2}{2J^2}\sum_l \int d^2k \;\, l\, f_l^0 \left(\partial_{k_x}\boldsymbol{\gamma} \times \partial_{k_y}\boldsymbol{\gamma}\right)\cdot\bar{\boldsymbol{m}} \tag{31}$$

with $\bar{\boldsymbol{m}} = \int d^2\boldsymbol{r}\hat{m}(\boldsymbol{r})/V$ and f_l^0 evaluated in the absence of λ. The term is proportional to the magnetization $\boldsymbol{M}$ and the corresponding resistivity scaling is unchanged: $\rho_{xy}^{\mathrm{AHE}} \sim (\lambda/E_F)^2(a/\ell)^2$. The AHE contribution may appear to have contributions from all occupied states. However, like all semi-classical transport coefficients, it can be re-written as an integral over the Fermi surface (38).

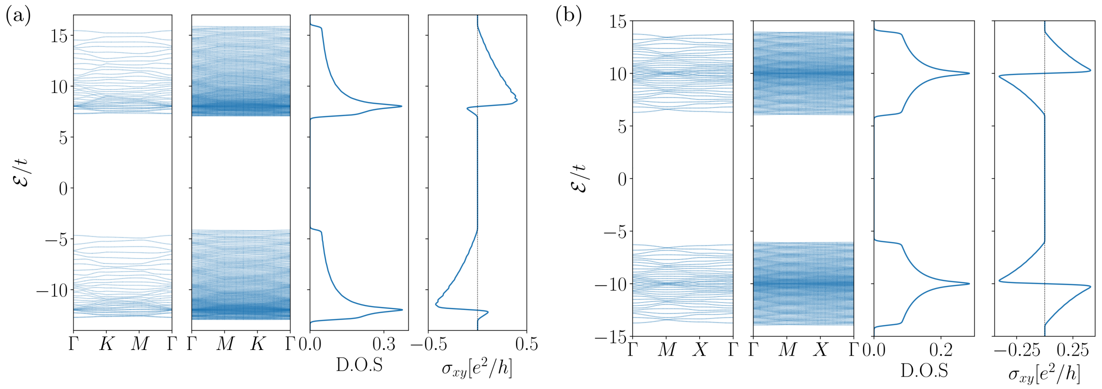

Figure S1: Hall Conductivity. Hall conductivity from exact diagonalization for a skyrmion crystal with (a) triangle and (b) square lattice with strong coupling $J/t = 10$. The first panel shows the bands for a skyrmion unit cell with $L_s/a = 6$. The skyrmion potential causes band folding between momentum eigenstates. The number of bands increases on increasing the lattice resolution of the skyrmion, as seen in the second panel with $L_s/a = 14$. The resulting density of states (D.O.S.) and Hall conductivity are shown in the last two panels.

The topological Hall contributions is completely unchanged and equation (13) remains completely valid with $\mathcal{E}_\pm(\boldsymbol{k})$ in $\mathcal{K}_l(\mu)$ given above.

The contribution $\delta\sigma_{xy}$ remains first order in spatial derivatives such that it remains a boundary contribution to the Hall effect and vanishes for periodic magnetic textures. Similiar to the case of Rashba spin-orbit interaction, in the presence of Dresselhaus spin-orbit coupling

$$\boldsymbol{\gamma}(\boldsymbol{k}) \cdot \boldsymbol{\sigma} = a(k_x\sigma_x - k_y\sigma_y) \tag{32}$$

and a free electronic dispersion, $\delta\sigma_{xy} \sim \lambda(\partial_{r_x} m_y(\mathbf{r}) + \partial_{r_y} m_x(\mathbf{r}))$ and can be interpreted as arising from an effective magnetic field (29).

Kubo formula calculation

We now turn to the limit when $a < L_s \ll \ell = \infty$. We use a tight binding model with magnetic unit cell area $\sim (L_s/a)^2$ and calculate the Hall conductance using the TKNN Kubo formula. The results of the calculation are exact and contain information deriving from all the types of

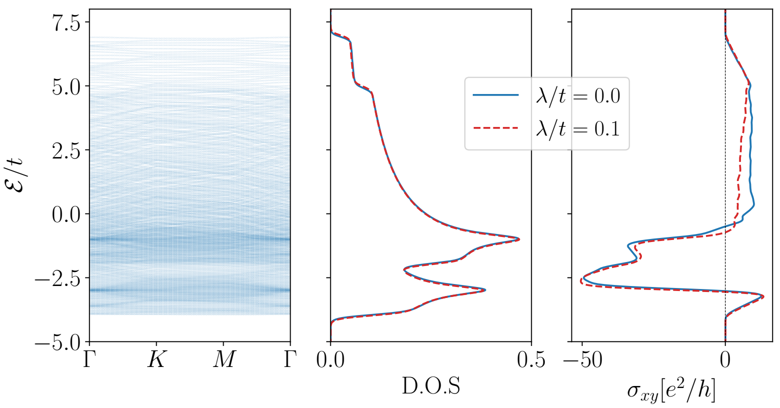

Figure S2: Effects of SOC. Panel (a) shows the band structure without SOC for small coupling with $J/t = 0.1$. There is no clear separation into states that align or anti-align with the texture. As λ is introduced, there is a small change in the DoS (as seen in panel (b)) and Hall conductivity (seen in panel (c)). The SOC-induced effects are significant only for large carrier densities.

contributions to the Hall effect.

We consider a tight-binding version of the continuum model

$$\mathcal{H} = -t \sum_{\langle \mathbf{i},\mathbf{j}\rangle,\sigma} c^\dagger_{\mathbf{i}\sigma} c_{\mathbf{j}\sigma} - i\lambda \sum_{\langle \mathbf{i},\mathbf{j}\rangle,\sigma,\sigma'} c^\dagger_{\mathbf{i}\sigma} \left[\mathbf{r}_{ij} \times \mathbf{z} \cdot \boldsymbol{\sigma}\right]_{\sigma\sigma'} c_{\mathbf{j}\sigma'} - J \sum_{\mathbf{i},\sigma,\sigma'} c^\dagger_{\mathbf{i}\sigma} \left[\hat{\mathbf{m}}_i \cdot \boldsymbol{\sigma}\right]_{\sigma\sigma'} c_{\mathbf{i}\sigma'} \tag{33}$$

where the vector field $\hat{\mathbf{m}}_{\mathbf{i}}$ models a discrete version of a skyrmion

$$\mathbf{m}_{\mathbf{i}} = \begin{pmatrix} \sin\left(2\pi \mathbf{i}\cdot\mathbf{a}_1\right) \\ \sin\left(2\pi \mathbf{i}\cdot\mathbf{a}_2\right) \\ \cos\left(2\pi \mathbf{i}\cdot\mathbf{a}_1\right) + \cos\left(2\pi \mathbf{i}\cdot\mathbf{a}_2\right) + 1 \end{pmatrix}, \quad \hat{\mathbf{m}}_{\mathbf{i}} = \frac{\mathbf{m}_{\mathbf{i}}}{\sqrt{\mathbf{m}_{\mathbf{i}}\cdot\mathbf{m}_{\mathbf{i}}}} \tag{34}$$

with winding number $+1$. Here $\mathbf{a}_1$ and $\mathbf{a}_2$ are the lattice vectors for the skyrmion lattice and $\mathbf{i}$ labels a position inside the skyrmion unit cell. The corresponding magnetic Brillouin Zone (MBZ) is spanned by vectors $\mathbf{b}_1$ and $\mathbf{b}_2$ that satisfy $\mathbf{a}_i \cdot \mathbf{b}_j = 2\pi\delta_{i,j}$. These vectors permit a momentum representation

$$c_{\mathbf{k}\sigma} = \frac{1}{\sqrt{N_{uc}}} \sum_{\mathbf{k}\in\text{MBZ}} e^{i\mathbf{k}\cdot\mathbf{i}} c_{\mathbf{i}\sigma} \tag{35}$$

with $\mathbf{k}$ taken from a $N_k \times N_k$ BZ mesh. The Bloch Hamiltonian which is then diagonalized to find the energies and wavefunctions

$$\mathcal{H}(\mathbf{k})|u_{n,\mathbf{k}}\rangle = \epsilon_n(\mathbf{k})|u_{n,\mathbf{k}}\rangle. \tag{36}$$

The wave-functions lead to the Berry curvature

$$\Omega_n(\mathbf{k}) = -2\mathrm{Im}\langle \partial_{k_x} u_{n,\mathbf{k}} | \partial_{k_y} u_{n,\mathbf{k}} \rangle \tag{37}$$

that is then combined with the TKNN formula to calculate the Hall conductivity

$$\sigma_{xy} = -\frac{e^2}{\hbar}\frac{1}{V}\int_{\text{MBZ}} \frac{d^2\mathbf{k}}{(2\pi)^2} \sum_{n=1}^{N_b} \Omega_n(\mathbf{k})\ \Theta(\mu - \epsilon_n(\mathbf{k})). \tag{38}$$

Finally, we replace the integral by a discrete sum

$$\frac{1}{V}\int_{\text{MBZ}} \frac{d^2\mathbf{k}}{(2\pi)^2} \longrightarrow \frac{1}{\mathcal{V} N_k^2} \sum_{\mathbf{k}\in\text{MBZ}} \tag{39}$$

where $\mathcal{V} = |\mathbf{a}_1 \times \mathbf{a}_2| = \sqrt{3}L_s^2/2$ is the area of the skyrmion unit cell. The carrier density is defined as

$$na^2 = \frac{1}{N_s^2 N_k^2} \sum_{n,\mathbf{k}\in\text{MBZ}} \Theta(\mu - \epsilon_n(\mathbf{k})) \tag{40}$$

so that it varies between 0 and 2 (see Fig. S1).

REFERENCES AND NOTES

1. N. Nagaosa, Y. Tokura, Topological properties and dynamics of magnetic skyrmions. *Nat. Nanotechnol.* **8**, 899–911 (2013).

2. M. Lee, W. Kang, Y. Onose, Y. Tokura, N. P. Ong, Unusual hall effect anomaly in MnSi under pressure. *Phys. Rev. Lett.* **102**, 186601 (2009).

3. A. Neubauer, C. Pfleiderer, B. Binz, A. Rosch, R. Ritz, P. G. Niklowitz, P. Böni, Topological hall effect in the *a* phase of MnSi. *Phys. Rev. Lett.* **102**, 186602 (2009).

4. N. Kanazawa, Y. Onose, T. Arima, D. Okuyama, K. Ohoyama, S. Wakimoto, K. Kakurai, S. Ishiwata, Y. Tokura, Large topological hall effect in a short-period helimagnet MnGe. *Phys. Rev. Lett.* **106**, 156603 (2011).

5. Y. Li, N. Kanazawa, X. Z. Yu, A. Tsukazaki, M. Kawasaki, M. Ichikawa, X. F. Jin, F. Kagawa, Y. Tokura, Robust formation of skyrmions and topological hall effect anomaly in epitaxial thin films of MnSi. *Phys. Rev. Lett.* **110**, 117202 (2013).

6. J. C. Gallagher, K. Y. Meng, J. T. Brangham, H. L. Wang, B. D. Esser, D. W. McComb, F. Y. Yang, Robust zero-field skyrmion formation in FeGe epitaxial thin films. *Phys. Rev. Lett.* **118**, 027201 (2017).

7. A. S. Ahmed, J. Rowland, B. D. Esser, S. R. Dunsiger, D. W. McComb, M. Randeria, R. K. Kawakami, Chiral bobbers and skyrmions in epitaxial FeGe/Si(111) films. *Phys. Rev. Materials* **2**, 041401 (2018).

8. A. S. Ahmed, A. J. Lee, N. Bagués, B. A. McCullian, A. M. A. Thabt, A. Perrine, P.K. Wu, J. R. Rowland, M. Randeria, P. C. Hammel, D. W. McComb, F. Yang, Spin-hall topological hall effect in highly tunable pt/ferrimagnetic-insulator bilayers. *Nano Lett.* **19**, 5683–5688 (2019).

9. Q. Shao, Y. Liu, G. Yu, S. K. Kim, X. Che, C. Tang, Q. L. He, Y. Tserkovnyak, J. Shi, K. L. Wang, Topological Hall effect at above room temperature in heterostructures composed of a magnetic insulator and a heavy metal. *Nat. Electron.* **2**, 182–186 (2019).

10. R. Karplus, J. M. Luttinger, Hall effect in ferromagnetics. *Phys. Rev.* **95**, 1154–1160 (1954).

11. N. Nagaosa, Anomalous hall effect—A new perspective. *J. Phys. Soc. Jpn.* **75**, 042001 (2006).

12. N. Nagaosa, J. Sinova, S. Onoda, A. H. MacDonald, N. P. Ong, Anomalous hall effect. *Rev. Mod. Phys.* **82**, 1539–1592 (2010).

13. D. Xiao, M.-C. Chang, Q. Niu, Berry phase effects on electronic properties. *Rev. Mod. Phys.* **82**, 1959–2007 (2010).

14. J. Smit, The spontaneous hall effect in ferromagnetics i. *Physica* **21**, 877–887 (1955).

15. L. Berger, Influence of spin-orbit interaction on the transport processes in ferromagnetic nickel alloys, in the presence of a degeneracy of the 3d band. *Physica* **30**, 1141–1159 (1964).

16. Y. Yao, L. Kleinman, A. H. MacDonald, J. Sinova, T. Jungwirth, D.-S. Wang, E. Wang, Q. Niu, First principles calculation of anomalous hall conductivity in ferromagnetic bcc fe, *Phys. Rev. Lett.* **92**, 037204 (2004).

17. J. Ye, Y. B. Kim, A. J. Millis, B. I. Shraiman, P. Majumdar, Z. Tešanović, Berry phase theory of the anomalous hall effect: Application to colossal magnetoresistance manganites. *Phys. Rev. Lett.* **83**, 3737–3740 (1999).

18. P. Bruno, V. K. Dugaev, M. Taillefumier, Topological hall effect and berry phase in magnetic nanostructures. *Phys. Rev. Lett.* **93**, 096806 (2004).

19. G. Tatara, H. Kawamura, Chirality-driven anomalous hall effect in weak coupling regime. *J. Phys. Soc. Jpn.* **71**, 2613–2616 (2002).

20. M. Onoda, G. Tatara, N. Nagaosa, Anomalous hall effect and skyrmion number in real and momentum spaces. *J. Phys. Soc. Jpn.* **73**, 2624–2627 (2004).

21. N. Nagaosa, X. Z. Yu, Y. Tokura, Gauge fields in real and momentum spaces in magnets: Monopoles and skyrmions. *Phil. Trans. R. Soc. A.* **370**, 5806–5819 (2012).

22. K. Nakazawa, M. Bibes, H. Kohno, Topological hall effect from strong to weak coupling. *J. Phys. Soc. Jpn.* **87**, 033705 (2018).

23. A. Matsui, T. Nomoto, R. Arita, Skyrmion-size dependence of the topological hall effect: A real-space calculation. *Phys. Rev. B* **104**, 174432 (2021).

24. K. W. Kim, H. W. Lee, K. J. Lee, M. D. Stiles, Chirality from interfacial spin-orbit coupling effects in magnetic bilayers. *Phys. Rev. Lett.* **111**, 216601 (2013).

25. K. Hamamoto, M. Ezawa, N. Nagaosa, Quantized topological hall effect in skyrmion crystal. *Phys. Rev. B* **92**, 115417 (2015).

26. C. A. Akosa, A. Takeuchi, Z. Yuan, G. Tatara, Theory of chiral effects in magnetic textures with spin-orbit coupling. *Phys. Rev. B* **98**, 184424 (2018).

27. F. R. Lux, F. Freimuth, S. Blügel, Y. Mokrousov, Engineering chiral and topological orbital magnetism of domain walls and skyrmions. *Commun. Phys.* **1**, 60 (2018).

28. H. Ishizuka, N. Nagaosa, Spin chirality induced skew scattering and anomalous hall effect in chiral magnets. *Sci. Adv.* **4**, eaap9962 (2018).

29. C. A. Akosa, H. Li, G. Tatara, O. A. Tretiakov, Tuning the skyrmion hall effect via engineering of spin-orbit interaction. *Phys. Rev. Applied* **12**, 54032 (2019).

30. S. S. Zhang, H. Ishizuka, H. Zhang, G. B. Halász, C. D. Batista, Real-space Berry curvature of itinerant electron systems with spin-orbit interaction. *Phys. Rev. B* **101**, 024420 (2020).

31. F. R. Lux, F. Freimuth, S. Blügel, Y. Mokrousov, Chiral hall effect in noncollinear magnets from a cyclic cohomology approach. *Phys. Rev. Lett.* **124**, 096602 (2020).

32. J. Bouaziz, H. Ishida, S. Lounis, S. Blügel, Transverse transport in two-dimensional relativistic systems with nontrivial spin textures. *Phys. Rev. Lett.* **126**, 147203 (2021).

33. J. Ziman, *Electrons and Phonons* (Oxford University Press, 2007).

34. F. Freimuth, R. Bamler, Y. Mokrousov, A. Rosch, Phase-space berry phases in chiral magnets: Dzyaloshinskii-moriya interaction and the charge of skyrmions. *Phys. Rev. B* **88**, 214409 (2013).

35. D. J. Thouless, M. Kohmoto, M. P. Nightingale, M. den Nijs, Quantized hall conductance in a two-dimensional periodic potential. *Phys. Rev. Lett.* **49**, 405–408 (1982).

36. Y. Tokura, N. Kanazawa, Magnetic skyrmion materials. *Chem. Rev.* **121**, 2857–2897 (2020).

37. T. Yokoyama, Absence of hall effect due to Berry curvature in phase space. *Sci. Rep.* **11**, 12065 (2021)

38. F. D. M. Haldane, Berry curvature on the Fermi surface: Anomalous Hall effect as a topological Fermi-liquid property. *Phys. Rev. Lett.* **93**, 206602 (2004).
